# Analysis of QTLs on heading date based on single segment substitution lines in rice (*Oryza Sativa* L.)

**DOI:** 10.1038/s41598-018-31377-7

**Published:** 2018-09-05

**Authors:** Haitao Zhu, Yun Li, Jiayan Liang, Xin Luan, Pan Xu, Shaokui Wang, Guiquan Zhang, Guifu Liu

**Affiliations:** 1 Guangdong Key Lab of Plant Molecular Breeding, South China Agricultural University, Guangzhou, 510642 P. R. China; 2 Shenzhen Agricultural Science and Technology Promotion Center, Shenzhen, 518055 P. R. China; 3 College of Computational Science, Zhongkai University of Agriculture and Engineering, Guangzhou, 510225 P. R. China

## Abstract

Single segment substitution lines (SSSLs) have been confirmed to be powerful tools to perform quantitative trait locus (QTL) analysis. This study illuminated the process and methods of QTL analysis with SSSLs on heading date (HD) in rice. QTL identification under two cropping seasons revealed 98 of 202 SSSLs associated with HD. A total of 22 QTLs were positioned in relative narrow regions on chromosomes by mrMLM.GUI software. QTL *qHd3-1* was precisely positioned at 4.4 cM on chromosome 3 by a secondary F2 population. Through SSSL pyramiding, double segment substitution lines were constructed and used to analyze epistatic interactions of digenic loci. Epistatic effects for three pairs of QTLs were estimated, indicating the interactions of QTL *qHd3-1* with other QTLs detected and the role to enhance the expression of early ripening or restraining of late flowering QTLs. Additionally, analysis of QTL in different environments provided information about the stability of HD QTLs. This type of research points out the way to excavate favorable genes for design breeding.

## Introduction

Heading date (HD) is a key agronomical determinant for current varieties of cultivated rice to adapt to specific cultivation regions and cropping seasons^1^. For example, typical short-day rice cultivars (*Oryza sativa* L.), which originated in tropical areas near the equator, might fail to suit for growing in high latitudes mainly since their flowering is hampered by the change in day length. Appropriate heading date is therefore the pre-requisite for ensuring maturity of grain and attaining the desired yield in rice under certain environmental conditions. Development of early-flowering or photoperiod-insensitive cultivars is a major objective in rice breeding programs^2^. Numerous genetic studies suggested that the inheritance of heading date is of polygenic nature, and genes were expressed or suppressed in close interaction with environmental factors such as day length and temperature etc. and other genes^3^.

How to analyze polygenic traits as heading date? Recent progress in the molecular technology and statistical methodology has already provided us efficient means for mapping quantitative trait loci (QTLs)^2^. By conventional mapping populations, such as mainly F2s, BC1F1s, RILs (recombinant inbred lines) or DHLs (doubled haploid lines), QTLs contributing to heading date in *Oryza* species had been intensively explored and mapped to their residing chromosomal regions^2,4^. However, these populations, in which multiple genetic factors on the whole genome segregate simultaneously, cannot be used as the materials for the precise mapping of QTLs. Moreover, it would be more difficult to determinate the true genetic actions of the QTLs and to differentiate QTL effects from background noise^5,6^.

Near-isogenic lines (NILs) developed by sequential backcrossing were widely used to accurate genetic analysis of genes because the NILs populations made the genetic background consistent^6,7^. This type of populations has been employed for QTL analysis on numerous traits in various crops. However, after the detection of putative QTLs with major effects, the construction of special QTL NILs was laborious and time consuming. Moreover, it might be impossible to achieve NILs of QTLs with relatively minor effects because QTL-NILs depend on QTLs detected in advance from the primary populations^8^.

An alternative strategy to analyze QTLs was proposed that the population of single segment substitution lines (SSSLs) was constructed first, and then QTL analysis was conducted by this population or the secondary SSSL populations developed^7,9^. SSSLs have the features similar to NILs but are easier to be developed than NILs. An additional advantage is the population of SSSLs can provide complete coverage of the genome, which makes QTL analysis surveyed on the whole genome. Through trait comparison between one of SSSLs and the recipient parent, QTLs on the substituted segments for measured traits can be conveniently identified even though they have minor effects^5^. Further fine mapping of QTLs identified can be conducted by construction of segregating populations obtained from crossing one of SSSLs and their recurrent parent^9^. Epistatic analysis between QTLs was well done via analysis of aggregation lines of SSSLs^10^.

A library of 1563 SSSLs on the recipient parent of HJX74 (Hua-jing-xian 74) has been constructed, and then the large-scale QTL analyses have been done on almost all interesting traits using the library. However, SSSLs used mostly carried with larger substitution segments, which might failure to position target QTLs precisely. Recent development of mapping software mrMLM (multi-locus random-SNP-effect mixed linear model) and QTL.gCIMapping (genome-wide composite interval mapping) could position QTLs adjusting to various populations and finely map QTLs^11,12^. In this study, HD was selected as one model trait to be investigated in order to describe the processes and methods by using SSSLs in our lab. It is showed that this is a good way to identify, isolate and evaluate the beneficial genes, and could provide useful information for breeding by design.

## Results

### Phenotypic Variation of HD

Table 1 showed the phenotypic variation of HJX74 and the SSSL population on HD in the spring and the fall, 2016, respectively. Averagely, HJX74 needed 87.43 days to flowering over the spring and the fall with the standard deviation of 9.15 days. Significant difference with the *t*-value of 11.35 between seasonal environments was observed on HD for HJX74. Comparing to those in the spring, environmental factors in the fall would largely reduce days to flowering by 32.85 days for HJX74. Similar case occurred for the SSSL population. For the population, HD was 87.83 days averaged over two seasons with the range from 53.75 to 126.65 days. Climate factors in the spring delayed flowering by about 33 days comparing to the fall. The SSSL population did not appear significantly different from HJX74 on HD. There were almost consistent variations of HD for HJX74 and the SSSL population across different seasons, indicating the interaction of genotype by environment was minor.

**Table 1:** Descriptives of heading date for Hua-jing-xian 74 (HJX74) and single segment substitution line (SSSL) population in the spring and the fall.

Environment	HJX74			SSSL population			
	Mean (days)	SD (days)	N	Mean (days)	SD (days)	Min (days)	Max (days)
The spring	103.85	10.96	201	104.33	6.42	83.30	126.65
The fall	71.00	6.89	197	70.99	4.15	53.75	92.60
Average of both	87.43	9.15	397	87.83	17.55	53.75	126.65

### QTL Identifications

Of 202 SSSLs presented in this trial (Supplementary Table S1), a total of 98 SSSLs was tested with significant differences compared to HJX74 on HD (Table 2). These substitution segments distributed on all the twelve chromosomes in various numbers from 2 to 17 for each chromosome. 50 SSSLs were detected to be significantly differed from HJX74 in only one seasonal environment. The remaindering 48 SSSLs existed significantly with the same directions of effects across the two environments. There were 42 and 29 SSSLs with significantly positive effects in the spring and in the fall, respectively. Effect values estimated among SSSLs ranged from −20.6 to 22.8 days in the spring and from −17.3 to 21.6 days in the fall, respectively. Most of SSSLs showed different effects of HD across the two environments, implying these effects were environmental sensitive (Table 2).

**Table 2:** Phenotypic and effect values (days) on heading date (hd) for Hua-jing-xian 74 (HJX74) and single segment substitution lines (SSSLs) with significant effects in the spring or the fall, respectively.

SSSL^a^	hd1	hd2	effect1		effect2^b^		SSSL	hd1	hd2	effect1		effect2	
HJX74	103.9	71											
s1-1	101.9	73.85	−2.0^c^		2.85	*,^d^	s6-11	121.85	90.9	17.95	**	19.9	**
s1-6	102.55	68.25	−1.35		−2.75	*	s6-12	118.9	92.6	15	**	21.6	**
s1-8	100	67.4	−3.9	**	−3.6	**	s6-14	123.45	79.2	19.55	**	8.2	**
s1-9	99.6	70.35	−4.3	**	−0.65		s6-17	109.85	77.7	5.95	**	6.7	**
s1-10	99.95	67.25	−3.95	**	−3.75	**	s6-20	107.9	72.8	4	**	1.8	
s1-15	99.6	68.55	−4.3	**	−2.45		s6-22	100.05	68.7	−3.85	**	−2.3	
s1-17	100.6	68.85	−3.3	*	−2.15		s6-23	101.2	69.15	−2.7	*	−1.85	
s1-20	104.55	73.85	0.65		2.85	*	s6-24	108.2	75.3	4.3	**	4.3	**
s2-2	99	69.65	−4.9	**	−1.35		s6-25	107.85	74.7	3.95	**	3.7	**
s2-6	100.35	71.7	−3.55	*	0.7		s6-29	111.1	70.5	7.2	**	−0.5	
s2-7	99.4	68.9	−4.5	**	−2.1		s7-1	100.3	68.15	−3.6	**	−2.85	*
s2-8	99.55	68.15	−4.35	**	−2.85	*	s7-4	110.2	74.1	6.3	**	3.1	*
s2-11	99.7	68.4	−4.2	**	−2.6	*	s7-5	100.8	70.95	−3.1	*	−0.05	
s2-12	100.15	64.95	−3.75	**	−6.05	**	s7-7	107.55	75.6	3.65	**	4.6	**
s2-13	98.25	62.45	−5.65	**	−8.55	**	s8-1	108.55	73.65	4.65	**	2.65	*
s2-14	100.4	67.8	−3.5	*	−3.2	*	s8-2	100.45	70.3	−3.45	**	−0.7	
s2-15	100.9	73.2	−3	*	2.2		s8-4	118.7	79.4	14.8	**	8.4	**
s2-16	95.25	66.2	−8.65	**	−4.8	**	s8-5	119.7	79.1	15.8	**	8.1	**
s2-17	109.65	72.2	5.75	**	1.2		s8-6	121.5	80.25	17.6	**	9.25	**
s3-1	88.35	64.15	−15.55	**	−6.85	**	s8-7	114.6	76.3	10.7	**	5.3	**
s3-2	97.8	62.95	−6.1	**	−8.05	**	s8-8	120.9	79.05	17	**	8.05	**
s3-3	83.3	53.75	−20.6	**	−17.25	**	s8-9	124.2	81.65	20.3	**	10.65	**
s3-4	92.5	65.1	−11.4	**	−5.9	**	s8-10	120.95	79.9	17.05	**	8.9	**
s3-5	96.8	63.95	−7.1	**	−7.05	**	s8-11	121.3	77.45	17.4	**	6.45	**
s3-6	97.75	67.4	−6.15	**	−3.6	**	s8-12	122.2	79.7	18.3	**	8.7	**
s3-7	96.9	62.7	−7	**	−8.3	**	s8-13	124.7	—	20.8	**		
s3-8	91.3	63.9	−12.6	**	−7.1	**	s8-14	115.1	84.9	11.2	**	13.9	**
s3-9	100.8	70.8	−3.1	*	−0.2		s8-15	101.45	67.65	−2.45		−3.35	*
s3-14	100.55	70.3	−3.35	**	−0.7		s8-19	106.8	73.15	2.9	*	2.15	
s3-16	97.25	65.85	−6.65	**	−5.15	**	s8-20	111.1	70.45	7.2	**	−0.55	
s3-18	101.95	68.4	−1.95		−2.6	*	s8-21	99.1	65.2	−4.8	**	−5.8	**
s3-22	97.5	70.45	−6.4	**	−0.55		s9-4	101.15	69.3	−2.75	*	−1.7	
s3-23	100.5	70.05	−3.4	*	−0.95		s9-6	100.4	69.5	−3.5	*	−1.5	
s3-24	101	71.6	−2.9	*	0.6		s10-3	110.9	72.8	7	**	1.8	
s3-27	99.95	63.9	−3.95	**	−7.1	**	s10-4	108.3	73.75	4.4	**	2.75	*
s4-2	102.65	68.15	−1.25		−2.85	*	s10-7	109.65	—	5.75	**		
s4-3	107.45	70.55	3.55	*	−0.45		s10-10	106.95	72.25	3.05	*	1.25	
s4-5	106.75	71.1	2.85	*	0.1		s10-11	109.1	74.8	5.2	**	3.8	**
s4-13	—	79.45			8.45	**	s10-12	110.15	74.05	6.25	**	3.05	*
s4-15	107.1	71.5	3.2	*	0.5		s10-13	107.15	71.35	3.25	*	0.35	
s5-2	102.55	68.35	−1.35		−2.65	*	s10-15	107.8	74.8	3.9	**	3.8	**
s5-3	102.5	64.6	−1.4		−6.4	**	s11-1	88.65	60.95	−15.25	**	−10.05	**
s5-5	102.4	68.4	−1.5		−2.6	*	s11-5	106.7	71.15	2.8	*	0.15	
s5-7	100.85	68.35	−3.05	*	−2.65	*	s11-6	105.65	73.65	1.75		2.65	*
s6-3	100.75	69.5	−3.15	*	−1.5		s11-10	97	64.5	−6.9	**	−6.5	**
s6-5	109.95	75.55	6.05	**	4.55	**	s11-11	99.6	69.45	−4.3	**	−1.55	
s6-7	126.65	—	22.75	**			s12-5	100.85	70.85	−3.05	*	−0.15	
s6-9	125.55	—	21.65	**			s12-6	106.6	75.4	2.7	*	4.4	*
s6-10	124.45	—	20.55	**			s12-7	100.8	69.7	−3.1	*	−1.3	

^a^si-j represented *jth* SSSL on chromosome *i*. ^b^hd1 and effect1, hd2 and effect2 indicated the phenotypic and effect values on HD for each SSSL in the spring and the fall, respectively. ^c^The negative sign in the table indicated to shorten HD. ^d, *^ and ^**^ showed the significance at the 0.05 and 0.01 levels, respectively.

### QTL Positions

**Figure 1:**
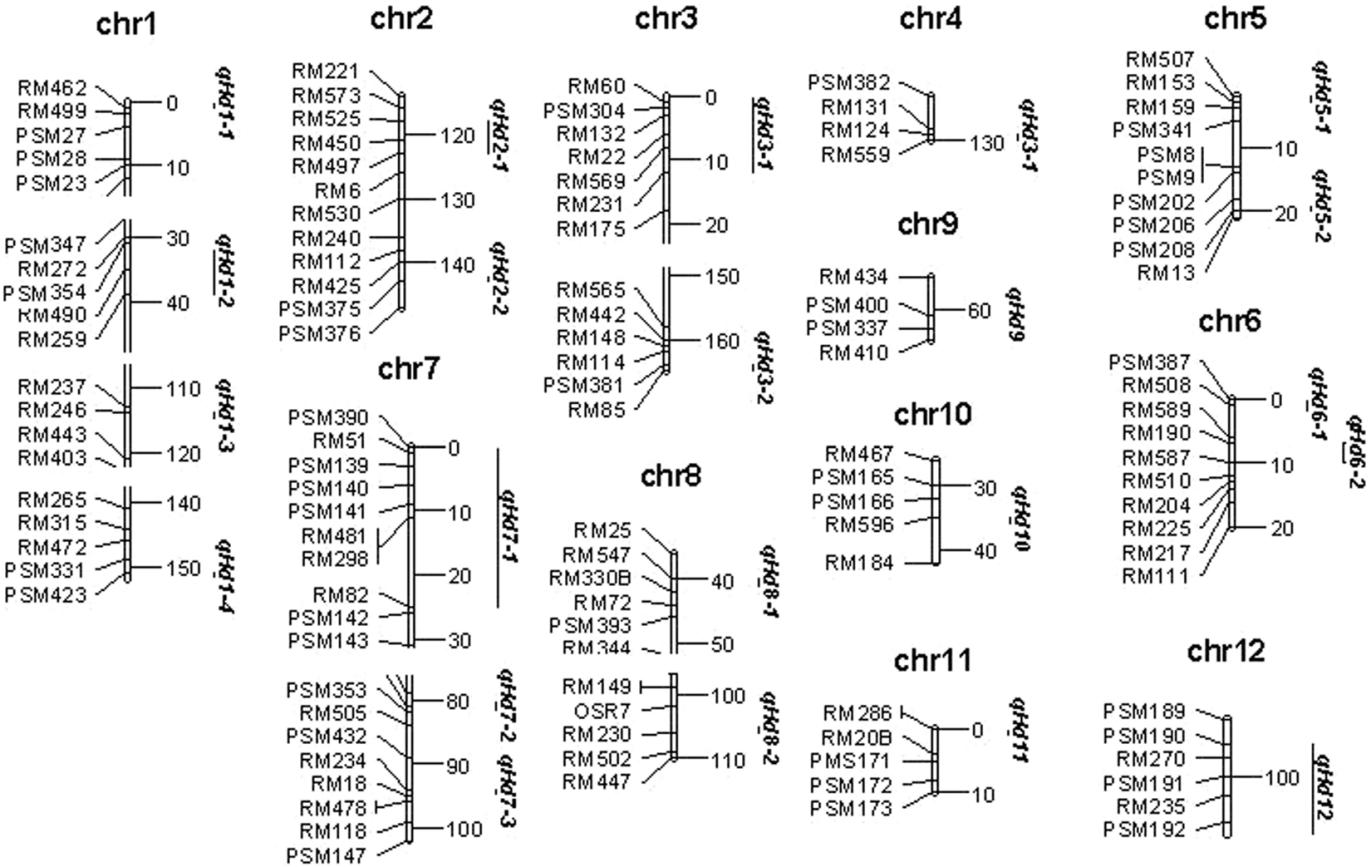
Distributions of QTLs positioned on heading date by the software of mrMLM.GUI on chromosomes (chr). Chr was the abbreviation of chromosome. Markers and genetic positions (cM) appeared at the left and the right of chromosomes, respectively. Points or vertical lines at the right of chromosomes represented QTLs.

Analysis of SSSLs could identify whether QTLs on target traits were on substitution segments or not. Since most of substitution segments existed among those SSSLs were large in length, it is difficult to locate QTLs in smaller marker intervals on these segments via SSSLs comparison. It is possible to position target QTLs by mrMLM.GUI software. We analyzed the data derived from HJX74 and all 202 SSSLs by this software, and then a total number of 22 QTL on HD was positioned within their resided regions (Fig. 1). Since most QTLs involved in multiple SSSLs, QTL effects were estimated by averaging over effects of participation SSSLs (Table 3). For example, QTL *qHd1-1* was located next to marker RM462, just involving in one SSSL, thus the effect of *qHd1-1* was estimated by the effect of the SSSL. However, QTL *qHd1-2* might involve in four participation SSSLs, s1-6, s1-8, s1-9 and s1-10, its effect was estimated as the average effect over the four SSSLs. It was interesting to note that some SSSLs might carry with two or more QTLs. For instance, SSSLs of s2-12 and s2-13 might include two QTLs *qHd2-1* and *qHd2-2* since their substitution segments covered the whole region of the two QTLs. Unfortunately, some SSSLs as s2-2, s2-6, s2-7 and s2-8 etc. were detected with significant effects, but their corresponding QTLs were not positioned. On the other hand, some QTLs positioned didn’t show significant effects on some of their corresponding SSSLs. For example, QTL *qHd1-1*, involving in SSSL of s1-2, didn’t exhibit significant effect on the SSSL.

**Table 3:** QTL effects estimated by averaging over effects of participation single segment substitution lines (SSSLs) on heading date (HD) in the spring and the fall.

QTL^a^	Marker	Position (cM)^b^	SSSL involved^c^	Effect estimated (days)^d^	
				The spring	The fall
*qHd1-1*	RM462	0.5	s1-1	−2.0^e^	2.9
*qHd1-2*	RM490–RM259	32.4–38.8	s1-6, s1-8, s1-9, s1-10	−3.4	−2.7
*qHd1-3*	RM246	114.1	s1-15, s1-17	−3.8	−2.3
*qHd1-4*	PSM423	151.0	s1-20	0.7	2.9
*qHd2-1*	RM525–RM497	118.1–122.8	s2-11, s2-12, s2-13	−4.5	−5.7
*qHd2-2*	PSM375	142.5	s2-12–s2-17	−3.1	−3.2
*qHd3-1*	RM60–RM231	0.5–11.5	s3-1–s3-9	−9.9	−7.1
*qHd3-2*	PSM381	164.4	s3-27	−4.0	−7.0
*qHd4*	RM559	129.6	s4-13, s4-15	3.2	4.5
*qHd5-1*	RM153	3.0	s5-2, s5-3	−1.4	−2.7
*qHd5-2*	PSM208	19.5	s5-3, s5-5, s5-7	−2.0	−3.9
*qHd6-1*	RM508	1.4	s6-3, s6-5	1.5	1.5
*qHd6-2*	RM190–RM587	7.4–10.4	s6-3, s6-7, s6-9–s6-12, s6-14, s6-29	15.2	9.5
*qHd7-1*	RM51–RM82	0.8–24.8	s7-1	−3.6	−2.9
*qHd7-2*	RM70–PSM353	80.5–81.9	s7-4, s7-5, s7-7	2.3	2.6
*qHd7-3*	RM234–RM18	93.9–94.7	s7-7	3.7	4.6
*qHd8-1*	RM547	40.2	s8-4–s8-14	16.5	8.8
*qHd8-2*	RM230	105.7	S8-19, s8-20	5.1	0.8
*qHd9*	PSM400	60.8	s9-4, s9-6	−3.1	−1.6
*qHd10*	RM596	35.1	s10-3, s10-4	5.7	2.3
*qHd11*	RM286	0.3	s11-1	−15.3	−10.1
*qHd12*	PSM190PSM193	95.1–109.2	s12-5, s12-6, s12-7	−1.2	1.0

^a^QTL was nominated by *qHd* followed chromosomal number or -serial number. ^b^Position (cM) indicated the positions of QTL on chromosomes. ^c^si-j represented *jth* SSSL on chromosome *i*. ^d^QTL effects were showed by the effects estimated by averaging over SSSLs involved. ^e^The negative sign in the table indicated to shorten HD.

### Fine Mapping for QTL *qHd3-1*

**Figure 2:**
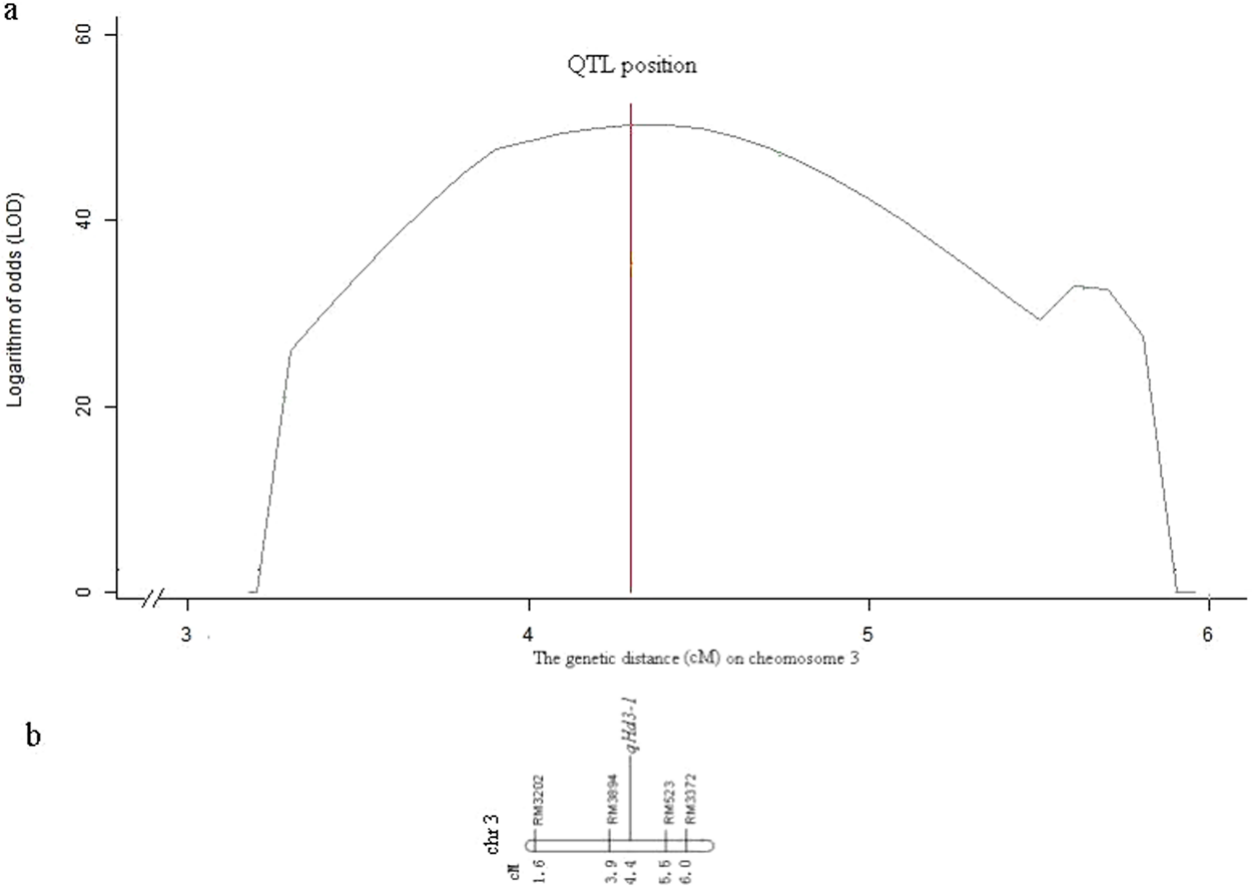
Fine position of QTL *qHd3-1* on chromosome 3 mapped by the software of QTL.gCIMapping. (**a**) Genetic distance–LOD curve. (**b**) Fine position of QTL *qHd3-1* on chromosome 3. Markers and QTL appeared above the chromosome, and their corresponding positions were ranged below the chromosome.

In the experiments, SSSL s3-3 showed a considerable reduction on HD relative to the control, being −20.6 days in the spring and −17.3 days in the fall, respectively. The effects were attributed to a single QTL *qHd3-1* that was positioned between RM60 and RM231 (Fig. 1 and Table 3). To obtain fine location of this QTL, we conducted fine mapping in the secondary generation progenies of s3-3 crossed with HJX74. A total of 197 individual plants and 4 microsatellite markers were investigated for separating ratios in the secondary F2 population, and then phenotypic data on HD and genotypic data on makers (Supplementary Table S2) were analyzed by QTL.gCIMapping.GUI software. The result indicated that QTL *qHd3-1* was precisely determined at 4.4 cM on chromosome 3, being in marker interval of RM3894–RM523 (Fig. 2).

### Epistatic Interactions between QTLs

To detect epistatic interactions between QTLs, two SSSLs were aggregated into a DSSL by MAS, and then the comparison of phenotypic performance among SSSLs, DSSL and HJX 74 on HD was conducted under the spring and the fall in 2016, respectively. Based on the effects estimated from SSSLs and DSSLs, epistatic effects between QTL *qHd3-1* on s3-2 and each of three QTLs, *qHd6-1* on s6-5 *qHd6-2* on s6-17 and *qHd8-2* on s8-20, were calculated (Table 4). For example, the epistatic effect between QTLs *qHd3-1* and *qHd6-1* was estimated by −5.8 days, e.g. the residual effect of DSSL effect (−5.8 days) departed from the algebraic sum of two SSSL effects (being −6.1 days and 6.1 days, respectively) in the spring (Table 2). Significant epistatic effects were detected between QTLs surveyed, but they were significantly different across environments. An important finding was that epistatic interaction occurred under the spring took a more important role than that under the fall on HD. Another was that all three pairs of epistatic effects promoted heading under both environments. The reasonable explanation was that early flowering QTL *qHd3-1* enhanced the expression of early ripening or restrained late flowering QTLs via epistatic interaction.

**Table 4:** Effects and epistatic effects estimated between QTL *qHd3-1* and one of three QTLs, *qHd6-1*, *qHd6-2* and *qHd8-2*, on heading date (HD) under the spring and the fall (days).

QTL aggregated^a^	DSSL^c^	Effect estimated (days)^e^		Epistatic effects estimated (days)^f^	
		The spring	The fall	The spring	The fall
*qHd3-1/qHd6-1* ^b^	s3-2/s6-5^d^	−5.8^*g^	−5.5^*h^	−5.8^*^	−2.1
*qHd3-1/qHd6-2*	s3-2/s6-17	−6.9^**^	−6.6^**^	−6.8^*^	−5.2^*^
*qHd3-1/qHd8-2*	s3-2/s8-20	−5.3^*^	−7.0^**^	−6.4^*^	1.8

^a^QTL aggregated denoted two target QTL that were used in aggregation. ^b^QTL was nominated by *qHd* followed chromosomal number or -serial number. ^c^DSSL (double substitution segment line) was developed from two SSSLs by MAS. ^d^si-j represented *jth* SSSL on chromosome *i*. ^e^Effect estimated was calculated by the genetic difference between DSSL and the control. ^f^Epistatic effect estimated equaled to the effect of DSSL subtracted the sum of two SSSL effects. ^g^The negative sign in the table indicated to shorten HD. ^h, *^ and ^**^ denoted the significance at the 0.05 and 0.01 levels, respectively.

## Discussion

### The processes of QTL analysis

A library of 1563 SSSLs on the recipient parent of HJX74 and donor parents of 26 worldwise varieties has been constructed^13^. Subsequently, the large-scale QTL identifications have been done for almost all interesting traits using these SSSLs^14–16^. Meanwhile, some SSSLs were selected as experimental materials to develop secondary populations such as F2 for QTL fine mapping and to aggregate SSSLs for epistatic analysis^8,17–22^ and design breeding^14^. This paper took data of HD evaluated from two cropping seasons in 2016 as an example to explain the process and validity of QTL analysis via SSSLs. A total of 202 SSSLs were used in the experiment, 98 of which were detected with HD QTLs (Table 2). Consistency of these QTLs was further tested by mrMLM mapping software, and a total of 22 QTLs were summarized and positioned in smaller regions on chromosomal substitution segments (Fig. 1 and Table 3). Fine mapping for one QTL (*qHd3-1*) was conducted in a secondary F2 population. Precise position of this QTL on chromosome was determined at 4.4 cM on chromosome 3 through linkage analysis between phenotypic data and 4 marker genotypic data of 197 plants by the QTL.gCIMapping.GUI software (Fig. 2). Epistatic effects for three pairs of digenic interactions were also estimated by the development of DSSLs (Table 4). Similar process of analysis occurred on almost all interesting characteristics for the final purpose of design breeding, and primary results to aggregate several favour single substitution segments into the recipient parent for improving target traits have been achieved in our previous studies^14^. This is a feasible scheme and has a great prospect. So far, we have basically finished the work of QTL identifications for various traits, and are executing the scheme of design breeding (unpublished data). The highlights in doing so are (1) being capable of getting large numbers of valuable materials with target gene or genes, and (2) to have avoided the labor- and time-intensive work to transfer genes. Other advantages for the construction of SSSLs to QTL analysis are able to obtain consistent QTLs, distinguish closely linked QTLs, and to analyze gene actions, such as various effect estimating and function analysis, via construction of pyramiding materials of SSSLs.

### QTL Fine Mapping

Traditionally, QTL fine mapping was conducted by a large number of plants or lines and genetic markers in a separating population. However, it is difficult to determine the precise locations and to prove the existence of QTLs with small effects^23^. NILs have many advantages for the genetic analysis, but they must be on the premise of QTL primary mapping by traditional mapping methods. In other words, QTLs must be detected first, and then QTL-NILs can be developed only. Thus, construction of NILs is labor- and time-intensive, and still be limited by initial QTLs detected by conventional methods^2^. Oppositely, it is easy that SSSLs were constructed first, and then were used to detect QTLs. Once a SSSL was detected with QTL controlling a trait, the material can be used to further QTL analysis such as fine mapping. This paper described two ways for QTL fine mapping. One is by construction of SSSL population to adapt to the method of mrMLM^11^. The other is by construction of secondary mapping population such as F2 population, which separates only in the target region on chromosome under consistent genetic background, to performance general QTL mapping^12^. From 202 SSSL population, several QTL were precisely positioned next to corresponding markers (Fig. 1 and Table 3). From a secondary F2 population, QTL *qHd3-1* was finely located at the position of 4.4 cM on chromosome 3 (Fig. 2). These results clearly demonstrated the accuracy of QTL fine mapping by using SSSL materials.

### Epistatic Interaction

SSSLs also make it possible to evaluate epistatic interactions between QTLs^10^. Pairs of digenic interactions among QTLs were also estimated via DSSLs in this study. Interaction between QTL *qHd3-1* and *qHd6-2* occurred strongly in the both cropping seasons, clearly suggesting that the pair of epistatic interaction was environmental stable. The remaindering two pairs of interactions were environmental sensitive since their significance occurred only in the spring season. All three pairs of epistatic interactions detected reduced days to heading, which perhaps is one of important genetic mechanisms for early ripening variety (Table 4). The result observed was consistent with the conclusion of Eshed and Zamir^10^. Digenic interactions between QTL *qHd3-1* and other HD QTL were also analyzed, and similar cases were detected (unpublished data). These results clearly demonstrated the role of QTL *qHd3-1* played in epistatic interactions with different types of QTL. The existence of QTL *qHd3-1* might restrain the expressing of late flowering QTL (Table 4), but enhance the expressing of early ripening QTL with some differences across seasonal environments (unpublished data). Thus we suggest that QTL *qHd3-1* might be a locus as a regulator controlling the expressing of other QTL on HD. This finding has important practical values in design breeding. A more early ripening line can be expected when QTL *qHd3-1* with another early ripening QTL is aggregated together (unpublished data).

### QTL on HD

Heading date has been widely studied in genetics. Large numbers of QTL on HD were publicized on http://www.gramene.org/qtl/index.html. At least 15 QTL controlling flowering time in rice were identified, 8 of which has precisely been mapped by Rice Genome Research Program (RGP) in Japan^1,24^ In our group, numerous HD QTL were preliminarily identified by using SSSLs^8,18,20^. In this study, a total of 22 HD QTL was detected under two different cropping seasons (Table 3). Most QTL could repeatedly emerge on different SSSLs, indicating that these QTL are reliable. 3 QTL, *qHd2-2*, *qHd6-1 and qHd7-2*, were detected to have approximately consistent performance in both seasonal environments, suggesting that these QTL are environmental insensitive. The others might be environmental sensitive. An common phenomena is that all QTL, except for *qHd1-1* and *qHd12*, had the same direction of effects under two seasons, basically revealing the essence feature of these QTL on HD. Epistatic interaction is pervasive for quantitative traits in creature, it is also on HD. QTL *qHd3-1* was tested to frequently interact with other QTL detected (Table 4), and played the role to either enhance the expressing of early ripening QTL or restrain late flowering QTL. Thus it is necessary to identify the single locus effect and interaction effects for a specific QTL before it can be applied for aggregation. Once SSSLs are detected with favour HD QTL, they can be directly used as materials to improve HD for HJX74 via pyramiding breeding.

## Methods

### Plant Materials

The recipient HJX74 (an elite *indica* variety from South China) and 202 SSSLs from it were applied in analysis of QTLs on HD (Supplementary Table S1 and Supplementary Fig. S1)^11^. Analysis of molecular markers (more than 460 SSR) confirmed that each SSSL contained single substituted segment from a donor only, and all substitution segments of SSSLs were distributed on 12 chromosomes with the average size of 15.8 cM and the coverage rate of 97% over the whole rice genome^13^. A set of secondary F2 population derived from the cross between one SSSL with target QTL and HJX74, including 197 individual plants, was constructed and applied in QTL fine mapping^12^, in which marker genotypes produced by separating occurred only within substituted segment region (Supplementary Table S2). Three double substitution segment lines (DSSLs) were used to assist epistatic analysis accompany with their SSSL components. DSSLs were developed from the cross of two SSSLs, which were the homozygotes with two target substitution segments confirmed by MAS.

### Trials and Trait Evaluated

The field trials were conducted at the experimental farm, South China Agricultural University, Guangzhou, China, during two cropping seasons, the spring season (from March to July) and the fall season (from July to November) in 2016. In each trial, all materials mentioned above for different purposes were planted simultaneously according to the practice that seeds germinated before being sown in a seedling bed, and 20 days later seedlings were transplanted to a paddy field. A completely randomized block design was adopted with three replications, in which each plot consisted of four rows including 40 plants with one plant per hill spaced at 16.7 cm × 20.0 cm. The experiments were managed followed the local standard practices. Days-to-heading were scored from seeding to heading of first panicle for each plant. The average days to heading of 20 plants in the center of each plot were as input data for QTL analysis.

### QTL Analysis

For QTL analysis, QTL effects and epistatic effects between QTLs were estimated by ${\hat{G}}_{j}-{\hat{G}}_{0}$ and ${\hat{G}}_{ij}-{\hat{G}}_{i}-{\hat{G}}_{j}+{\hat{G}}_{0}$, which significance was tested by two-tail *t*-test at the 0.05 or 0.01 level of significance. Where, ${\hat{G}}_{i}\,or\,{\hat{G}}_{j},\,{\hat{G}}_{ij},\,{\hat{G}}_{0}$ were the estimates of genotypes SSSL, DSSL and HJX74, respectively. The effect estimates and their significant tests were performed by lm() function of R language (https://www.r-project.org/). For position of QTLs, the software of mrMLM.GUI^11^ was applied in data both HJX74 and all SSSLs. For fine mapping of QTLs, the software of QTL.gCIMapping.GUI was applied in data from the secondary F2 population (https://cran.r-project.org/web/packages/QTL.gCIMapping/index.html)^12^.

## Electronic supplementary material

Supplementary Materials
