# Supplementary material for: Analysis of QTLs on heading date based on single segment substitution lines in rice (Oryza Sativa L.)
Source: Sci Rep. 2018 Sep 5;8:13232. doi: 10.1038/s41598-018-31377-7 (PMC6125461; doi:10.1038/s41598-018-31377-7)
Supplement: Supplementary file 1 — Supplementary Materials [file 41598_2018_31377_MOESM1_ESM.pdf]

Analysis of QTLs on heading date based on single segment substitution lines in rice (*Oryza Sativa* L.)

Haitao Zhu[1#], Yun Li[1, 2#], Jiayan Liang[1#], Xin Luan[1], Pan Xu[3], Shaokui Wang[1*], Guiquan Zhang[1*], Guifu Liu[1*]

[1] Guangdong Key Lab of Plant Molecular Breeding, South China Agricultural University, Guangzhou 510642, P. R. China

[2] Shenzhen Agricultural Science and Technology Promotion Center, Shenzhen 518055, P. R. China

[3] College of Computational Science, Zhongkai University of Agriculture and Engineering, Guangzhou 510225, P. R. China

[*] Co-corresponding authors: Shaokui Wang, Guiquan Zhang and Guifu Liu

[#] Haitao Zhu, Yun Li and Jiayan Liang contributed equally to this work.

Phone: +86–20–85288311

Fax: +86–20–85288311

E-mail: guifuliu@scau.edu.cn

SUPPLEMENTARY MATERIALS

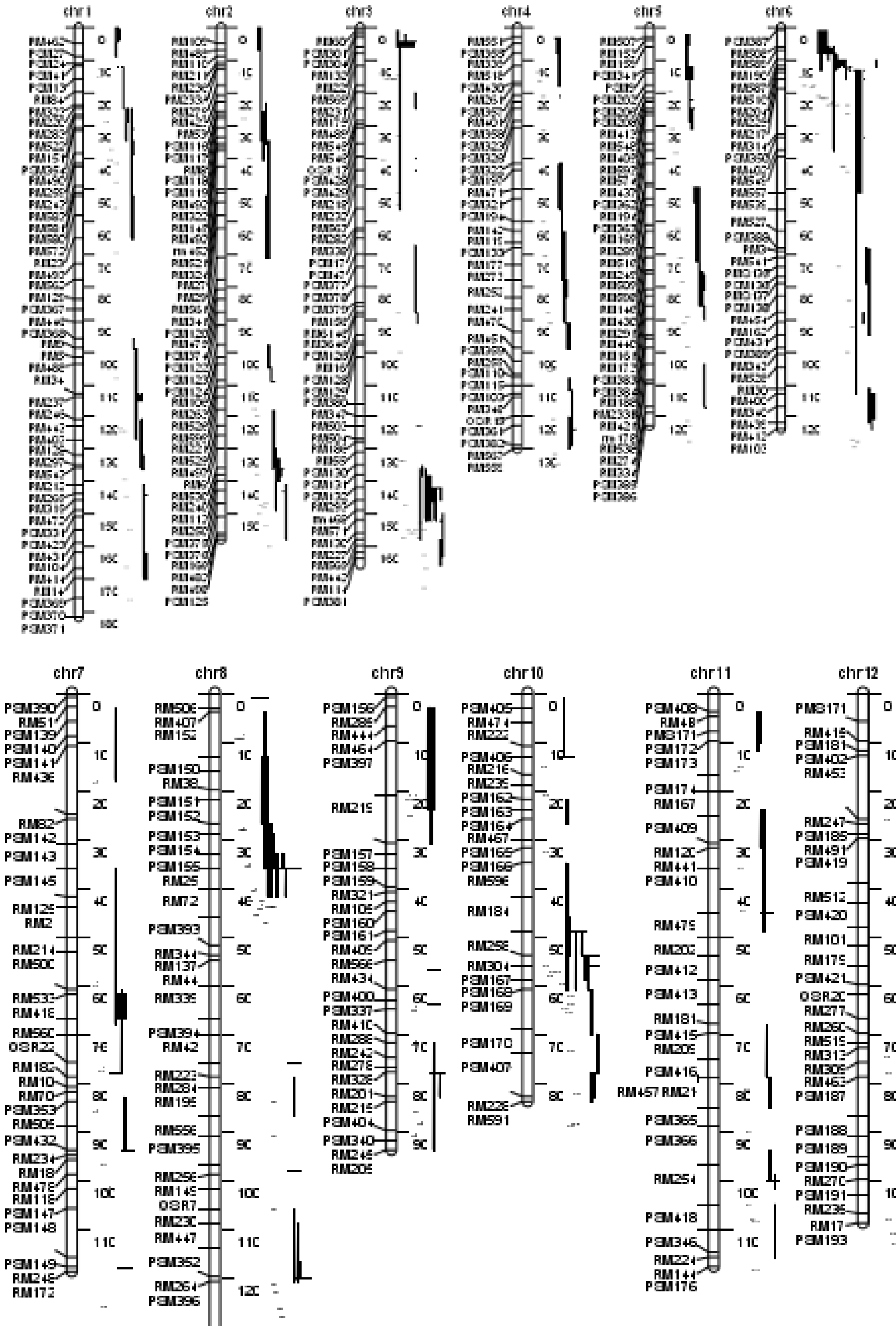

FIGURE S1 Distributions of single segments on the chromosomes (chr). Chr was the abbreviation of chromosome. Markers and genetic positions (cM) appeared at the left and the right of chromosomes, respectively. Points or vertical lines at the right of chromosomes represented substitution segments.

TABLE S1 Phenotypic values and effect values on heading date (hd) for Hua-jing-xian 74 (HJX74) and 202 single segment substitution lines (SSSLs) in the two cropping seasons (days)

SSSL[a]	hd1	hd2	effect1		effect2[b]		SSSL	hd1	hd2	effect1		effect2	
HJX74	103.9	71											
s1-1	101.9	73.85	-2.0[c]		2.85	*[d]	s6-4	104.15	68.8	0.25		-2.2	
s1-2	103.75	73.1	-0.15		2.1		s6-5	109.95	75.55	6.05	**	4.55	**
s1-3	102.8	69.65	-1.1		-1.35		s6-6	102.5	70.2	-1.4		-0.8	
s1-4	102	70.4	-1.9		-0.6		s6-7	126.65	-	22.75	**		
s1-5	103.9	72.45	0		1.45		s6-8	101.75	69.65	-2.15		-1.35	
s1-6	102.55	68.25	-1.35		-2.75	*	s6-9	125.55	-	21.65	**		
s1-7	102.8	72.15	-1.1		1.15		s6-10	124.45	-	20.55	**		
s1-8	100	67.4	-3.9	**	-3.6	**	s6-11	121.85	90.9	17.95	**	19.9	**
s1-9	99.6	70.35	-4.3	**	-0.65		s6-12	118.9	92.6	15	**	21.6	**
s1-10	99.95	67.25	-3.95	**	-3.75	**	s6-13	101.95	70.95	-1.95		-0.05	
s1-11	102.75	69.05	-1.15		-1.95		s6-14	123.45	79.2	19.55	**	8.2	**
s1-12	105.65	71.1	1.75		0.1		s6-15	104.4	71.45	0.5		0.45	
s1-13	101.6	70.3	-2.3		-0.7		s6-16	101.9	71	-2		0	
s1-14	101.7	69.95	-2.2		-1.05		s6-17	109.85	77.7	5.95	**	6.7	**
s1-15	99.6	68.55	-4.3	**	-2.45		s6-18	104.1	71.8	0.2		0.8	
s1-16	105.45	71.9	1.55		0.9		s6-19	102.55	68.9	-1.35		-2.1	
s1-17	100.6	68.85	-3.3	*	-2.15		s6-20	107.9	72.8	4	**	1.8	
s1-18	104.4	73.4	0.5		2.4		s6-21	101.55	69.45	-2.35		-1.55	
s1-19	103.65	72.65	-0.25		1.65		s6-22	100.05	68.7	-3.85	**	-2.3	
s1-20	104.55	73.85	0.65		2.85	*	s6-23	101.2	69.15	-2.7	*	-1.85	
s2-1	101.35	70.75	-2.55		-0.25		s6-24	108.2	75.3	4.3	**	4.3	**
s2-2	99	69.65	-4.9	**	-1.35		s6-25	107.85	74.7	3.95	**	3.7	**
s2-3	101.8	72.15	-2.1		1.15		s6-26	104.25	71.6	0.35		0.6	
s2-4	103.1	70	-0.8		-1		s6-27	103.9	70.55	0		-0.45	
s2-5	102.05	69.35	-1.85		-1.65		s6-28	101.75	70.65	-2.15		-0.35	
s2-6	100.35	71.7	-3.55	*	0.7		s6-29	111.1	70.5	7.2	**	-0.5	
s2-7	99.4	68.9	-4.5	**	-2.1		s7-1	100.3	68.15	-3.6	**	-2.85	*
s2-8	99.55	68.15	-4.35	**	-2.85	*	s7-2	101.25	70.95	-2.65		-0.05	
s2-9	103.85	71.65	-0.05		0.65		s7-3	102.5	69.85	-1.4		-1.15	
s2-10	104.45	69.35	0.55		-1.65		s7-4	110.2	74.1	6.3	**	3.1	*
s2-11	99.7	68.4	-4.2	**	-2.6	*	s7-5	100.8	70.95	-3.1	*	-0.05	
s2-12	100.15	64.95	-3.75	**	-6.05	**	s7-6	102.65	71.1	-1.25		0.1	
s2-13	98.25	62.45	-5.65	**	-8.55	**	s7-7	107.55	75.6	3.65	**	4.6	**
s2-14	100.4	67.8	-3.5	*	-3.2	*	s7-8	105.25	71.3	1.35		0.3	
s2-15	100.9	73.2	-3	*	2.2		s7-9	103.1	73.05	-0.8		2.05	
s2-16	95.25	66.2	-8.65	**	-4.8	**	s8-1	108.55	73.65	4.65	**	2.65	*

s2-17	109.65	72.2	5.75	**	1.2		s8-2	100.45	70.3	-3.45	**	-0.7	
s2-18	103.3	72.05	-0.6		1.05		s8-3	105.8	72.35	1.9		1.35	
s2-19	103.2	72.2	-0.7		1.2		s8-4	118.7	79.4	14.8	**	8.4	**
s2-20	103.35	72.1	-0.55		1.1		s8-5	119.7	79.1	15.8	**	8.1	**
s3-1	88.35	64.15	-15.55	**	-6.85	**	s8-6	121.5	80.25	17.6	**	9.25	**
s3-2	97.8	62.95	-6.1	**	-8.05	**	s8-7	114.6	76.3	10.7	**	5.3	**
s3-3	83.3	53.75	-20.6	**	-17.25	**	s8-8	120.9	79.05	17	**	8.05	**
s3-4	92.5	65.1	-11.4	**	-5.9	**	s8-9	124.2	81.65	20.3	**	10.65	**
s3-5	96.8	63.95	-7.1	**	-7.05	**	s8-10	120.95	79.9	17.05	**	8.9	**
s3-6	97.75	67.4	-6.15	**	-3.6	**	s8-11	121.3	77.45	17.4	**	6.45	**
s3-7	96.9	62.7	-7	**	-8.3	**	s8-12	122.2	79.7	18.3	**	8.7	**
s3-8	91.3	63.9	-12.6	**	-7.1	**	s8-13	124.7	-	20.8	**		
s3-9	100.8	70.8	-3.1	*	-0.2		s8-14	115.1	84.9	11.2	**	13.9	**
s3-10	103.05	71.15	-0.85		0.15		s8-15	101.45	67.65	-2.45		-3.35	*
s3-11	102.2	70.9	-1.7		-0.1		s8-16	102.2	72.5	-1.7		1.5	
s3-12	103.4	69.4	-0.5		-1.6		s8-17	102.15	70.2	-1.75		-0.8	
s3-13	101.25	68.95	-2.65		-2.05		s8-18	102.9	69.15	-1		-1.85	
s3-14	100.55	70.3	-3.35	**	-0.7		s8-19	106.8	73.15	2.9	*	2.15	
s3-15	101.6	70.4	-2.3		-0.6		s8-20	111.1	70.45	7.2	**	-0.55	
s3-16	97.25	65.85	-6.65	**	-5.15	**	s8-21	103.15	71.05	-0.75		0.05	
s3-17	104.75	68.9	0.85		-2.1		s8-22	99.1	65.2	-4.8	**	-5.8	**
s3-18	101.95	68.4	-1.95		-2.6	*	s9-1	104.25	72	0.35		1	
s3-19	103.05	68.9	-0.85		-2.1		s9-2	102.05	71.95	-1.85		0.95	
s3-20	102.55	69	-1.35		-2		s9-3	103.4	71.5	-0.5		0.5	
s3-21	101.85	70.85	-2.05		-0.15		s9-4	101.15	69.3	-2.75	*	-1.7	
s3-22	97.5	70.45	-6.4	**	-0.55		s9-5	104.6	69.85	0.7		-1.15	
s3-23	100.5	70.05	-3.4	*	-0.95		s9-6	100.4	69.5	-3.5	*	-1.5	
s3-24	101	71.6	-2.9	*	0.6		s9-7	101.9	70.55	-2		-0.45	
s3-25	101.8	68.9	-2.1		-2.1		s9-8	104.65	70.7	0.75		-0.3	
s3-26	103.75	72	-0.15		1		s9-9	104.75	71	0.85		0	
s3-27	99.95	63.9	-3.95	**	-7.1	**	s10-1	102.7	70.25	-1.2		-0.75	
s4-1	102.55	70.85	-1.35		-0.15		s10-2	101.85	69.5	-2.05		-1.5	
s4-2	102.65	68.15	-1.25		-2.85	*	s10-3	110.9	72.8	7	**	1.8	
s4-3	107.45	70.55	3.55	*	-0.45		s10-4	108.3	73.75	4.4	**	2.75	*
s4-4	103.35	70.9	-0.55		-0.1		s10-5	106.15	71.5	2.25		0.5	
s4-5	106.75	71.1	2.85	*	0.1		s10-6	103.4	71	-0.5		0	
s4-6	101.9	69.55	-2		-1.45		s10-7	109.65	-	5.75	**		
s4-7	102.85	71.65	-1.05		0.65		s10-8	101.2	69.65	-2.7		-1.35	
s4-8	102.1	71.75	-1.8		0.75		s10-9	106.05	71.55	2.15		0.55	
s4-9	101.8	70.55	-2.1		-0.45		s10-10	106.95	72.25	3.05	*	1.25	
s4-10	104.3	70.1	0.4		-0.9		s10-11	109.1	74.8	5.2	**	3.8	**
s4-11	105.1	68.8	1.2		-2.2		s10-12	110.15	74.05	6.25	**	3.05	*

s4-12	105.95	71.05	2.05		0.05		s10-13	107.15	71.35	3.25	*	0.35	
s4-13	-	79.45			8.45	**	s10-14	104.2	72.9	0.3		1.9	
s4-14	101.6	69.35	-2.3		-1.65		s10-15	107.8	74.8	3.9	**	3.8	**
s4-15	107.1	71.5	3.2	*	0.5		s10-16	104.15	71.8	0.25		0.8	
s5-1	103.25	69.25	-0.65		-1.75		s10-17	105.15	71.9	1.25		0.9	
s5-2	102.55	68.35	-1.35		-2.65	*	s11-1	88.65	60.95	-15.25	**	-10.05	**
s5-3	102.5	64.6	-1.4		-6.4	**	s11-2	102.8	70.35	-1.1		-0.65	
s5-4	104.8	70	0.9		-1		s11-3	105.65	69.85	1.75		-1.15	
s5-5	102.4	68.4	-1.5		-2.6	*	s11-4	106.05	71.7	2.15		0.7	
s5-6	101.65	70.2	-2.25		-0.8		s11-5	106.7	71.15	2.8	*	0.15	
s5-7	100.85	68.35	-3.05	*	-2.65	*	s11-6	105.65	73.65	1.75		2.65	*
s5-8	101.45	70	-2.45		-1		s11-7	102.3	71.7	-1.6		0.7	
s5-9	104.55	69.5	0.65		-1.5		s11-8	102.35	69.4	-1.55		-1.6	
s5-10	103.85	70.25	-0.05		-0.75		s11-9	105.9	72.05	2		1.05	
s5-11	102.7	69.65	-1.2		-1.35		s11-10	97	64.5	-6.9	**	-6.5	**
s5-12	104.85	71.9	0.95		0.9		s11-11	99.6	69.45	-4.3	**	-1.55	
s5-13	106.35	73.05	2.45		2.05		s12-1	101.25	68.85	-2.65		-2.15	
s5-14	102.8	71.3	-1.1		0.3		s12-2	102.95	68.5	-0.95		-2.5	
s5-15	102.1	71.1	-1.8		0.1		s12-3	104.25	70.7	0.35		-0.3	
s5-16	102.95	71.35	-0.95		0.35		s12-4	103.8	69.4	-0.1		-1.6	
s6-1	102.6	69.95	-1.3		-1.05		s12-5	100.85	70.85	-3.05	*	-0.15	
s6-2	103.15	71.45	-0.75		0.45		s12-6	106.6	75.4	2.7	*	4.4	*
s6-3	100.75	69.5	-3.15	*	-1.5		s12-7	100.8	69.7	-3.1	*	-1.3	

[a] si-j represented *jth* SSSL on chromosome i. [b] hd1 and effect1, hd2 and effect2 indicated the phenotypic values and effect values on HD for each SSSL in the spring and the fall, respectively. [c] The negative sign in the table indicated to shorten HD. [d] * and ** showed the significance at $P<0.05$ and 0.01, respectively

Table S2 Data derived from the secondary F2 population from $s3-3 \times HJX74$

pos（cM)[a]		1.6	3.9	5.5	6	pos（cM)		1.6	3.9	5.5	6
ind.	hd[b]	RM3202[c]	RM3894	RM523	RM3372	ind.	hd	RM3202	RM3894	RM523	RM3372
1	57	3[d]	3	3	3	100	62	2	2	2	2
2	58	2	2	2	2	101	62	2	2	2	2
3	58	3	3	3	3	102	62	2	2	2	2
4	58	3	3	3	3	103	62	3	3	3	3
5	58	3	2	2	2	104	62	2	2	2	2
6	58	2	2	2	2	105	62	2	2	2	2
7	58	2	2	2	2	106	62	3	3	3	3
8	59	2	2	2	2	107	62	2	2	2	2
9	59	3	3	2	2	108	62	0	2	2	2
10	59	3	3	3	3	109	62	2	2	2	2
11	59	3	3	3	3	110	62	2	2	2	2
12	59	2	2	3	3	111	62	1	3	3	3
13	59	2	2	2	2	112	62	2	2	2	2
14	59	2	2	2	2	113	62	3	3	3	3
15	59	2	2	2	2	114	62	3	3	3	3
16	59	3	3	3	3	115	62	2	2	2	2
17	59	2	2	2	2	116	62	2	2	2	2
18	60	3	3	3	3	117	62	3	3	3	3
19	60	3	3	3	3	118	62	2	2	2	2
20	60	3	3	3	3	119	62	3	2	2	2
21	60	2	2	2	2	120	62	3	3	3	3
22	60	2	2	2	2	121	62	3	3	3	3
23	60	2	2	2	2	122	62	0	2	2	2
24	60	3	3	3	3	123	62	2	2	3	3
25	60	2	2	2	2	124	62	2	2	2	2
26	60	2	2	2	2	125	63	2	2	2	2
27	60	1	1	1	1	126	63	3	3	3	3
28	60	2	2	2	2	127	63	2	2	2	2
29	60	2	2	2	2	128	63	0	2	2	2
30	60	3	3	3	3	129	63	2	2	2	2
31	60	3	3	3	3	130	63	2	2	2	2
32	60	2	2	2	2	131	63	2	2	2	2
33	60	3	3	3	3	132	63	2	2	2	2
34	60	3	3	3	3	133	63	2	2	2	2
35	60	1	1	1	1	134	63	2	2	2	2
36	60	2	2	2	2	135	63	3	3	3	3

37	60	3	3	3	3	136	63	0	2	2	2
38	60	2	2	2	2	137	63	2	2	2	2
39	60	2	2	2	2	138	64	2	2	2	2
40	60	2	2	2	2	139	64	2	2	2	2
41	60	1	2	2	2	140	64	2	2	2	2
42	60	2	2	2	2	141	64	2	2	2	2
43	60	3	3	3	3	142	64	2	2	2	2
44	60	3	3	3	3	143	64	2	2	2	2
45	60	2	2	2	2	144	64	2	2	2	2
46	60	2	2	2	2	145	64	2	2	2	2
47	60	1	1	1	1	146	65	2	2	2	2
48	60	3	3	3	3	147	65	2	2	2	3
49	60	2	2	2	2	148	65	2	2	2	2
50	60	2	2	2	2	149	65	2	2	2	2
51	60	2	2	2	2	150	65	2	2	2	2
52	60	2	2	2	2	151	65	2	2	2	2
53	60	0	3	3	3	152	65	2	2	2	2
54	60	3	3	3	3	153	65	2	2	2	2
55	60	2	2	2	2	154	65	3	3	3	3
56	60	3	3	3	3	155	65	2	2	2	2
57	60	2	2	2	2	156	66	2	2	2	2
58	60	3	3	3	3	157	66	2	2	2	2
59	60	3	3	3	3	158	70	1	1	1	1
60	61	2	2	2	2	159	70	1	1	0	1
61	61	2	2	2	2	160	70	0	1	1	1
62	61	3	3	3	3	161	70	0	1	1	1
63	61	2	2	2	2	162	71	1	1	1	1
64	61	3	3	3	3	163	71	1	1	1	1
65	61	3	3	3	3	164	71	1	1	1	1
66	61	3	3	3	3	165	71	1	1	1	1
67	61	2	2	2	2	166	71	1	1	1	2
68	61	1	2	2	2	167	71	1	1	1	1
69	61	2	2	2	2	168	71	1	1	1	1
70	61	2	2	2	2	169	72	1	1	1	1
71	61	2	2	2	2	170	72	1	1	1	1
72	61	2	2	2	2	171	72	1	1	1	1
73	61	2	2	2	2	172	72	1	1	1	1
74	61	3	3	3	3	173	72	1	1	1	1
75	61	2	2	2	2	174	73	1	1	1	2
76	61	3	3	3	3	175	73	1	1	1	1

77	61	2	2	2	2	176	73	1	1	1	1
78	61	2	2	2	2	177	73	1	1	1	1
79	61	3	3	3	3	178	73	2	1	1	1
80	61	2	2	2	2	179	73	1	1	1	1
81	61	2	2	2	2	180	73	1	1	1	1
82	61	2	2	2	2	181	73	2	1	1	1
83	61	2	2	2	2	182	73	2	1	1	1
84	61	2	2	2	2	183	73	1	1	1	1
85	61	2	2	2	2	184	74	1	1	1	1
86	62	2	2	2	2	185	74	1	1	1	1
87	62	3	3	3	3	186	74	1	1	1	1
88	62	2	2	2	2	187	74	1	1	1	1
89	62	2	2	2	2	188	74	1	1	1	1
90	62	2	2	2	2	189	74	1	1	1	1
91	62	3	3	3	3	190	75	1	1	1	1
92	62	0	2	2	2	191	75	1	1	1	1
93	62	3	3	3	3	192	75	1	1	1	1
94	62	2	2	2	2	193	75	1	1	1	1
95	62	2	2	2	2	194	76	1	1	1	1
96	62	3	2	2	1	195	76	1	1	1	1
97	62	2	2	2	1	196	77	1	1	1	1
98	62	2	2	2	2	197	77	1	1	1	1
99	62	3	3	3	3						

[a] Pos(cM) indicated the positions of markers on chromosomes. [b] ind. and hd represented the serial order and heading date, respectively. [c] Four SSR markers were named as RM3202, RM3894, RM523 and RM3372, respectively. [d] The numbers 1, 2 and 3 represented the genotypes of female parents, heterozygotes and male parents for each marker respectively, and 0 was absent.
